# Cuproptosis-related prognostic signatures predict the prognosis and immunotherapy in HCC patients

**DOI:** 10.1097/MD.0000000000034741

**Published:** 2023-08-25

**Authors:** Hong Peng, Zhaoxia Zou, Ziye Xiang, Xingjun Lu, Yiya Zhang, Xiaozhen Peng

**Affiliations:** a Medical School, Huanghe Science &Technology College, Zhengzhou, Henan, China; b School of Public Health & Laboratory Medicine, Hunan University of Medicine, Huaihua, Hunan, China; c Department of Dermatology, Xiangya Hospital, Central South University, Changsha, China; d Hunan Provincial Key Laboratory for Synthetic Biology of Traditional Chinese Medicine, Hunan University of Medicine, Huaihua, Hunan, China.

**Keywords:** cuproptosis-related, gene, lncRNA, immune, HCC

## Abstract

Cuproptosis, an unusual type of programmed cell death mechanism of cell death, involved the disruption of specific mitochondrial metabolic enzymes in the occurrence and development of tumors. However, it was still unclear how the relationship between cuproptosis-related genes (CRGs) may contribute to hepatocellular carcinoma (HCC) potential the prognosis of HCC remained limited. Here, the landscape of 14 CRGs in HCC was evaluated using the Cancer Genome Atlas and International Cancer Genome Consortium datasets. And then, 4 CRGs (ATP7A, MTF1, GLS, and CDKN2A) were screened for the construction of risk signatures for prognosis and drug therapy. The HCC patients with CRGs high-risk showed poor prognosis than those with low risk. Moreover, the CRGs risk signature was shown to be an independent prognostic factor and associated with the immune microenvironment in HCC. Meanwhile, we constructed and verified a prognostic model based on cuproptosis-related lncRNAs (Cr-lncRNAs). We obtained 291 Cr-lncRNAs and constructed Cr-lncRNA prognosis signature based on 3 key Cr-lncRNAs (AC026356.1, NRAV, AL031985.3). The Cr-lncRNA prognosis signature was also an independent prognostic factor and associated with the immune microenvironment in HCC. Finally, the drug sensitivity database showed that 8 candidate drugs related to CRGs signature and Cr-lncRNAs signature. In summary, we evaluated and validated the CRGs and Cr-lncRNAs as potential predictive markers for prognosis, immunotherapy, and drug candidate with the personalized diagnosis and treatment of HCC.

## 1. Introduction

Hepatocellular carcinoma (HCC) is the most prevalent type of liver cancer (90%) and the fourth leading cause of mortality among all cancers.^[1]^ Despite improvements in clinical outcomes via liver transplantation, percutaneous approaches, and hepatic resection,^[2,3]^ patients with HCC frequently have challenging prognostic predictions and experience cancer relapse.^[4]^ Hence, there is an urgent need to discover novel and reliable screening methods to establish more accurate diagnoses and effective treatments to improve prognosis.

Tsvetkov et al revealed that copper-dependent, regulated cell death is dependent on the binding of copper to lipoylated components of the tricarboxylic acid cycle in mitochondrial respiration, resulting in a novel pattern of cell death termed cuproptosis.^[5]^ Researchers have found that mitochondrial stress, loss of Fe-S cluster proteins, and aggregation of lipoylated mitochondrial enzymes, triggers cuproptosis.^[5]^ Accumulating evidence shows that copper ionophores are involve in intracellular copper accumulation, leading to copper-induced cell death.^[6]^ In recent years, cuproptosis has been identified as a mechanism of programmed cell death characterized by enhanced copper-induced cell death.^[7,8]^ Three components are essential for cuproptosis, copper importer (SLC31A1), lipoylation pathway (FDX1, LIAS, LIPT1, and DLD), and pyruvate dehydrogenase complex (DLAT, PDHA1, PDHB, MTF1, GLS, and CDKN2A).^[9]^ Recently, cuproptosis has emerged as a novel insight into the prediction and prognosis of cancer patients. Studies have shown that cuproptosis-related prognostic markers have the potential to predict overall survival (OS) and immune characteristics. The cuproptosis-related genes (CRGs) signature was built to predict OS in clear cell renal cell carcinoma.^[10]^ However, the landscape of CRGs and their prognostic role in HCC remain obscure. Therefore, we aimed to probe CRGs-based signature to estimate the prognosis of patients with HCC.

In the current study, CRGs and cuproptosis-related lncRNAs (Cr-lncRNAs) signatures were constructed to predict the prognosis of HCC. These 2 signatures are the independent predictors and are associated with mutation profiles, tumor microenvironment, and therapeutic response, with great promise for personalized treatment strategies.

## 2. Methods

### 2.1. Data

We downloaded the gene expression profiles and related clinical characteristics of HCC and normal tissues from the International Cancer Genome Consortium (ICGC), the Cancer Genome Atlas (TCGA), and GEO (GSE10140) databases. Of the 374 HCC tissues and 50 normal liver tissues from TCGA, 240 HCC tissues and 197 normal liver tissues from ICGC-LIRI-JP (Liver Cancer—RIKEN, JP), TCGA database was used to download the mutation data of HCC patients.

Fourteen CRGs, including 3 Copper importers, 5 lipoylation pathway-related genes, and 6 members of the pyruvate dehydrogenase complex, were collected from gene set enrichment analysis. The expression of CRGs was downloaded from TCGA and ICGC for differential expression analysis using R “edgeR” packages with *P* value < .05 and |log2FC| > 0.5. With the R package “clusterProfiler,” Gene Ontology and Kyoto Encyclopedia of Genes and Genomes were performed for function enrichment.

The varied characteristics of CRGs were performed using “RCircos” package. Mutation analysis was performed using R packages “maftools.”

### 2.2. Cuproptosis-related prognosis signature

The univariate Cox regression, Least absolute shrinkage and selection operator (LASSO), and multivariate Cox regression were used for constructing the risk model in the TCGA dataset (train) using “glmnet” package. Kaplan–Meier analysis and receiver operating characteristic (ROC) were used to evaluate the predictive ability of CRGs the signature using “survival” and “time ROC.”

Risk score = (exp Gene1 × coefficient gene1) + (exp Gene2 × coefficient gene2)+…+(exp GeneN × coefficient GeneN)

The ICGC datasets were used as validation datasets.

### 2.3. Independent prognostic analysis

The risk signature and clinical features from TCGA and ICGC datasets were applied to independent prognostic factors using univariate/multivariable Cox regression. Next, prognosis of patients with HCC was predicted using a nomogram. The predictive accuracy of the nomogram model was evaluated using ROC and calibration curves.

### 2.4. Immune microenvironment analysis

CIBERSORT, ESTIMATE, and single-sample gene set enrichment analyses were used for immune functions and immune infiltration. Immune cells and functions were analyzed between different clusters or risk groups.

The immune-related genes were downloaded from the IMMPORT database (ImmPort Portal), and the relationship between the CRGs cluster/signature was determined. Immune-related genes were analyzed using differential expression analysis and Pearson correlation analysis.

### 2.5. Identification of CRPs-related lncRNAs (Cr-lncRNA)

Cr-lncRNAs were obtained by calculating the correlation between CRPs, and the screening threshold was set at a correlation coefficient > 0.4 and *P* < .001.

### 2.6. Drug response analysis

The “pRRophetic” package was used to screen the drug response of patients with low-/high-risk. CellMiner was used to provide drug sensitivity to the CRGs signature genes. The immune therapy data were downloaded from the TCIA database (https://tcia.at/home) and the relationship between the risk score and immune therapy was analyzed using R “ggpubr” packages. We utilized Students *t* test to compare high/low groups’ differences.

### 2.7. Statistical analysis

R (version 4.1.1) was used for statistical analysis. We used the Wilcoxon test to compare difference between the 2 groups. The expression correlation was determined using person analysis.

## 3. Results

### 3.1. The landscape of cuproptosis-related genes in HCC

We first screened the expression and variation of 14 CRGs using TCGA databases. As shown Figure S1A, http://links.lww.com/MD/J505 (Supplemental Content, which illustrates the heatmap of CRGs expression in HCC), the expression of CRGs was compared between the HCC tissues and normal liver tissues. The copy number variation status and variations in CRGs were determined, and the results are showed in Figure S1B-1D, http://links.lww.com/MD/J505 (Supplemental Content, which selects CRGs in HCC). CDKN2A possessed the most frequent mutations. LIPT2 showed significant copy number amplification, while CDKN2A showed significant copy number deletion.

**Figure 1.**
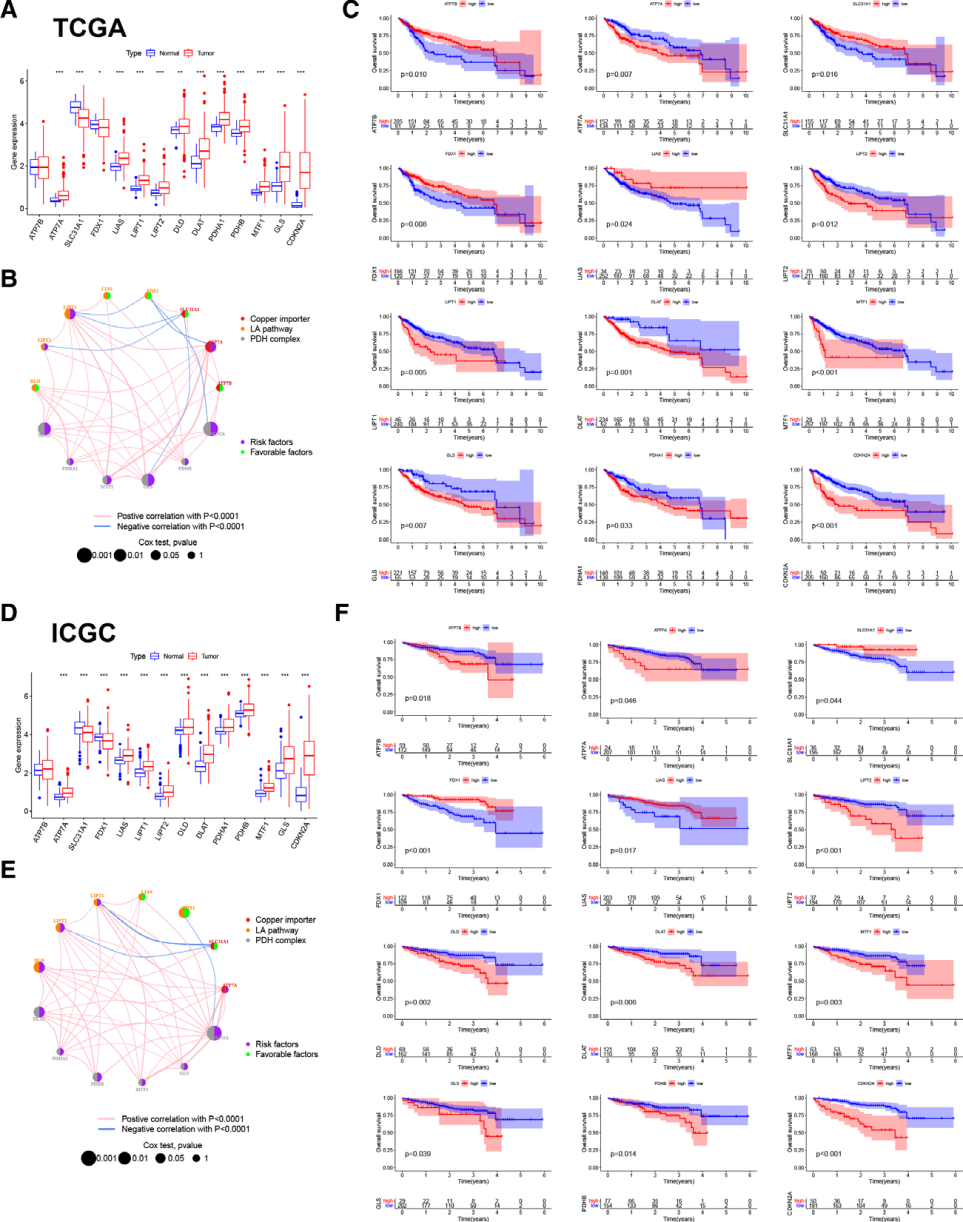
The expression and prognosis of CRGs in HCC from TCGA and ICGC datasets. The differently expression of CRGs in cancer tissues compared with normal liver tissues using TCGA dataset (A) and ICGC dataset (D). *P < .05, **P < .01, ***P < .001. The interaction between CRGs in HCC using TCGA dataset (B) and ICGC dataset (E). Green dots represented protective factors of prognosis, and purple dots represented risk factors of prognosis in the circle. The CRGs as Copper importer, LA pathway and PDH complex were marked with red, yellow and brown, respectively. The survival analysis of CRGs in HCC from TCGA dataset (C) and ICGC dataset (F). CRGs = cuproptosis-related genes, HCC = hepatocellular carcinoma.

We evaluated the expression and prognostic capability of these CRGs in HCC using TCGA and ICGC datasets (Fig. 1). Thirteen CRGs were differentially expressed between HCC and normal liver tissues in both TCGA (Fig. 1A) and ICGC (Fig. 1D) datasets. The expression of ATP7A, PDHA1, LIAS, LIPT1, GLS, LIPT2, DLD, DLAT, PDHB, MTF1, and CDKN2A was up-regulated, while the expression of SLC31A1, and FDX1 was down-regulated in HCC tissues compared with that in normal liver tissues. Figure 1B and E show the expression correlation among CRGs expression in HCC tissues from TCGA and ICGC datasets using Pearson correlation analysis. Next, we explored the prognostic role of CRGs in hepatoma carcinoma by dividing patients into low-and high-expression groups based on the median of CRGs expression. The low expression of ATP7A, LIPT2, DLAT, MTF1, GLS, and CDKN2A in patients with HCC was associated with poor OS, and the high expression of SLC31A1, FDX1, and LIAS in patients with HCC was associated with poor OS in both TCGA (Fig. 1C) and ICGC datasets (Fig. 1F).

### 3.2. CRGs prognostic signature of HCC

**Figure 2.**
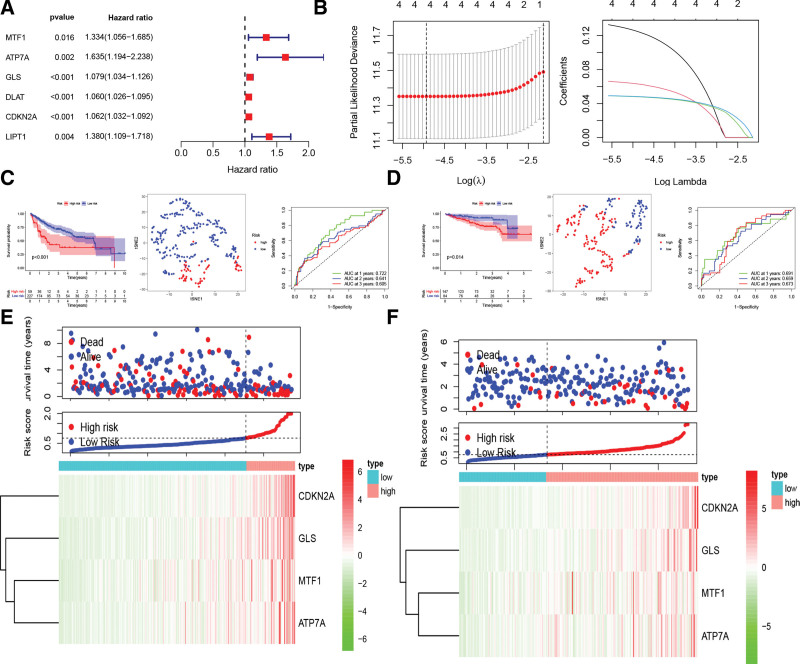
CRGs prognostic signature of HCC in the TCGA dataset and verified it in ICGC datasets. (A) The univariate Cox analysis. (B) The LASSO Cox regression analysis. (C) The survival analysis, the t-SNE analysis and the ROC curve of HCC patients with high/low-risk scores using TCGA dataset. (D) The survival analysis, the t-SNE analysis and the ROC curve of HCC patients with high/low-risk scores using ICGC dataset. The distribution of the risk score and the heatmap of CRGs signature genes using TCGA datasets (E) and ICGC datasets (F). CRGs = cuproptosis-related genes, HCC = hepatocellular carcinoma.

Univariate Cox and LASSO Cox regression analyses were used to construct the CRGs prognostic signature using the TCGA datasets. Six prognostic-related CRGs were identified using the univariate Cox analysis (Fig. 2A). To construct a CRGs prognostic signature model, we performed LASSO Cox regression analysis (Fig. 2B). The risk score = MTF1*0.12544 + ATP7A*0.0634 + GLS*0.0481 + CDKN2A*0.0483. Patients with HCC were divided into low and high-risk group using the best cutoff risk score in TCGA datasets (Fig. 2C). The results showed that high-risk HCC patients had a worse OS. The t-SNE analysis showed a high/low-risk group distribution, with an AUC of 0.722 for 1-year, 0.641 for 2-year, and 0.606 for 3-year survival, as revealed by the ROC curve in Figure 2C. Using the median risk score as the threshold, patients with HCC from the ICGC-LIRI-JP dataset were categorized into high- and low-risk categories (Fig. 2D). The prognosis, t-SNE, and ROC analyses of the CRGs signature were verified in the ICGC datasets (Fig. 2D). The survival status, risk score distribution and expression of 4 genes are presented. As shown in Figure 2E and F, the survival of HCC patients decreased when MTF1, ATP7A, GLS, and CDKN2A expression increased in both TCGA and ICGC datasets, which implied that those 5 genes were risk genes associated with poor prognosis of HCC patients.

In addition, GSVA analysis was performed to explore the differences in the participating KEGG pathways between the low-risk and high-risk groups. As shown in Figure S2 (see Fig. S2, http://links.lww.com/MD/J506, Supplemental Content, which illustrates GSVA analysis), the Th cell cycle pathway was up-regulated, and linoleic acid metabolism, arachidonic acid metabolism, and nitrogen metabolism were lower in the high-risk group than in the low-risk group using both TCGA and ICGC datasets. We next analyzed the prognosis of HCC patients with high/low-risk in different clinicopathological groups. Figure S3, http://links.lww.com/MD/J507 (Supplemental Content, which illustrates the survival analysis of HCC patients with high/low risk) shown that the OS of patients with stage I-II, T1-2, stage III-IV, T3-4, age <60 years, male sex, G1-2, M0, and N0 with high-risk was inferior to that of low-risk patients. Furthermore, we analyzed the genetic alterations between the high and low groups. The waterfall plot showed that the TP53 gene harbored the highest frequency of mutation, followed by TTN in high-risk patients (see Fig. S4A, http://links.lww.com/MD/J508, Supplemental Content, which illustrates the gene variation of HCC patients in high/low risk). Tumor mutation burden analysis showed a poor survival probability in HCC patients with a high mutation frequency of tumor mutation burden + high risk (H-TMB + high risk) (see Fig. S4B, http://links.lww.com/MD/J508, Supplemental Content, which illustrates the gene variation of HCC patients in high/low risk).

### 3.3. CRGs signature is an independent prognostic signature

**Figure 3.**
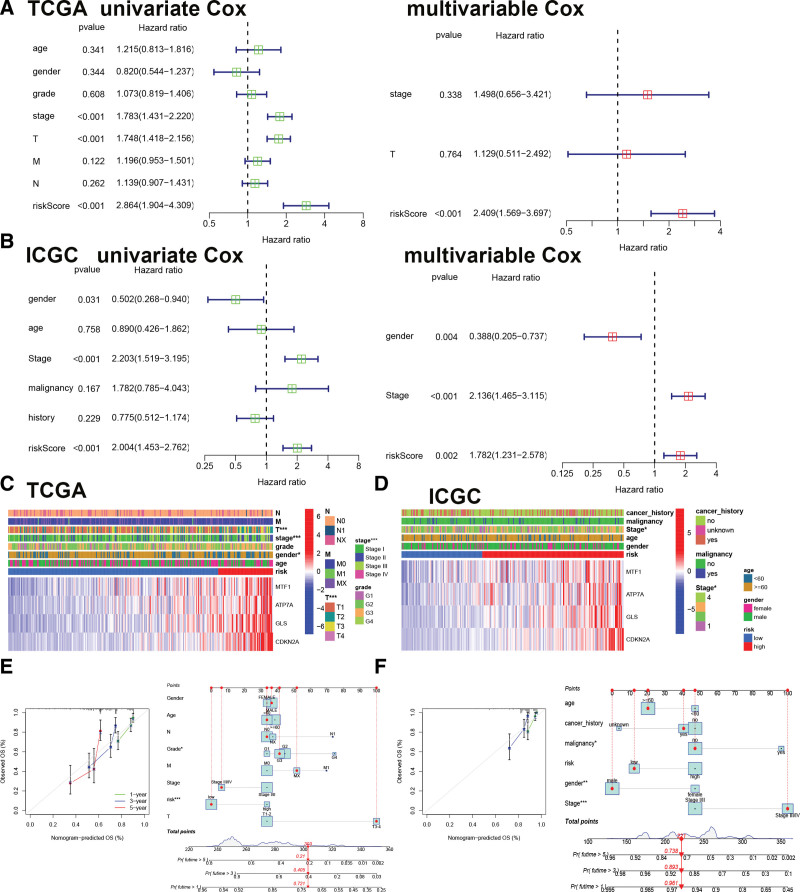
CRGs signature is an independent prognostic signature. (A) Univariate and multivariate analysis for TCGA dataset. (B) Univariate and multivariate analysis for ICGC dataset. (C) The relationship between risk genes expression and clinical characters in HCC using TCGA dataset. (D) The relationship between risk genes expression and clinical characters in HCC using ICGC dataset. (E) The nomogram model and calibration plots for predicted probability in TCGA dataset. (F) The nomogram model and calibration plots for predicted probability in ICGC dataset. CRGs = cuproptosis-related genes, HCC = hepatocellular carcinoma.

Data analysis was performed in multivariate and univariate modes to evaluate whether the CRGs signature was an independent prognostic factor for HCC. Figure 3A showed that the HR of the CRGs risk score was 2.864 (95%CI: 1.904–4.309) and 2.409 (95%CI: 1.569–3.697) in the univariate and multivariate analyses, respectively, indicating an independent prognostic factor in HCC using TCGA dataset. Similar results also were observed for the ICGC datasets (Fig. 3B). Figure 3C and D displayed a correlation between the risk score and clinical characteristics. The results showed that the CRGs signature was correlated with stage. To provide a quantitative instrument for the clinician, the risk score and clinicopathological features were collected to build a predictive nomogram, suggesting that the 1-, 3-, and 5-year OS rates could be assessed correspondingly perfectly compared with an ideal model utilizing TCGA and ICGC datasets (Fig. 3E and F).

### 3.4. CRGs signature is associated with immune microenvironment in HCC

**Figure 4.**
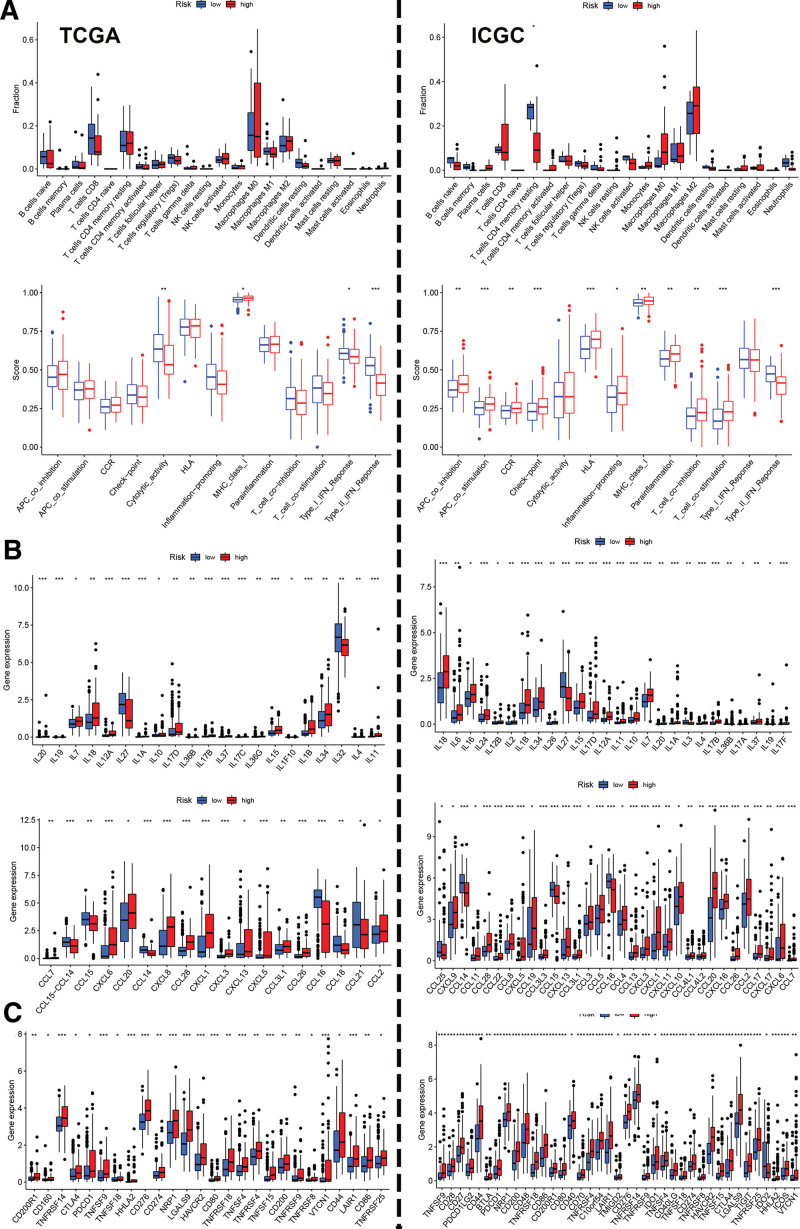
CRGs signature is associated with immune microenvironment in HCC. (A) The immune infiltration and immune function in high/low group in TCGA (left) and ICGC (right) datasets. (B) The cytokine and Chemokines expression in HCC patients with high/low-risk signature from TCGA (left) and ICGC (right) datasets. (C) The immune checkpoint expression in TCGA (left) and ICGC (right) datasets. CRGs = cuproptosis-related genes, HCC = hepatocellular carcinoma.

The immune microenvironment plays an essential role in HCC development and progression. We analyzed the differences in the immune microenvironment between low- and high-risk patients with HCC. As shown in Figure 4A, resting dendritic cells decreased in the high-risk group, and the Macrophages M0 and Mast cells resting evidently increased in the high-risk group in both the TCGA and ICGC datasets. Regarding immune function, the Type II IFN response was evidently decreased in the high-risk group (Fig. 4A). Immune infiltration using QUANTISEQ, CIBERSORT, and XCELL showed similar results Figure S5, http://links.lww.com/MD/J509 (Supplemental Content, which illustrates the different immune infiltration in high/low group). Next, we analyzed cytokine and chemokine expression in high- and low-risk groups. In the Figure 4B shown, IL7, IL18, IL12A, IL17D, IL15, IL1B, and IL34 were significantly up-regulated in the high-risk patients. CXCL6, CCL20, CCL28, CXCL1, CXCL3, CXCL13, CXCL5, CCL3L1, CCL26, and CCL2 were also significantly up-regulated in the high-risk group. Next, we explored checkpoint expression in HCC. Notably, the expression of immune checkpoints (including CD200R1, TNFRSF14, CTLA4, TNFSF9, TNFSF18, HHLA2, CD276, NRP1, LGALS9, HAVCR2, CD80, TNFRSF18, TNFSF4, TNFRSF4, CD200, TNFRSF9, TNFRSF8, VTCN1, CD44, LAIR1, CD86, and TNFRSF25) were significantly increased in the high risk-group (Fig. 4C).

### 3.5. Construction of Cr-lncRNAs model in HCC

**Figure 5.**
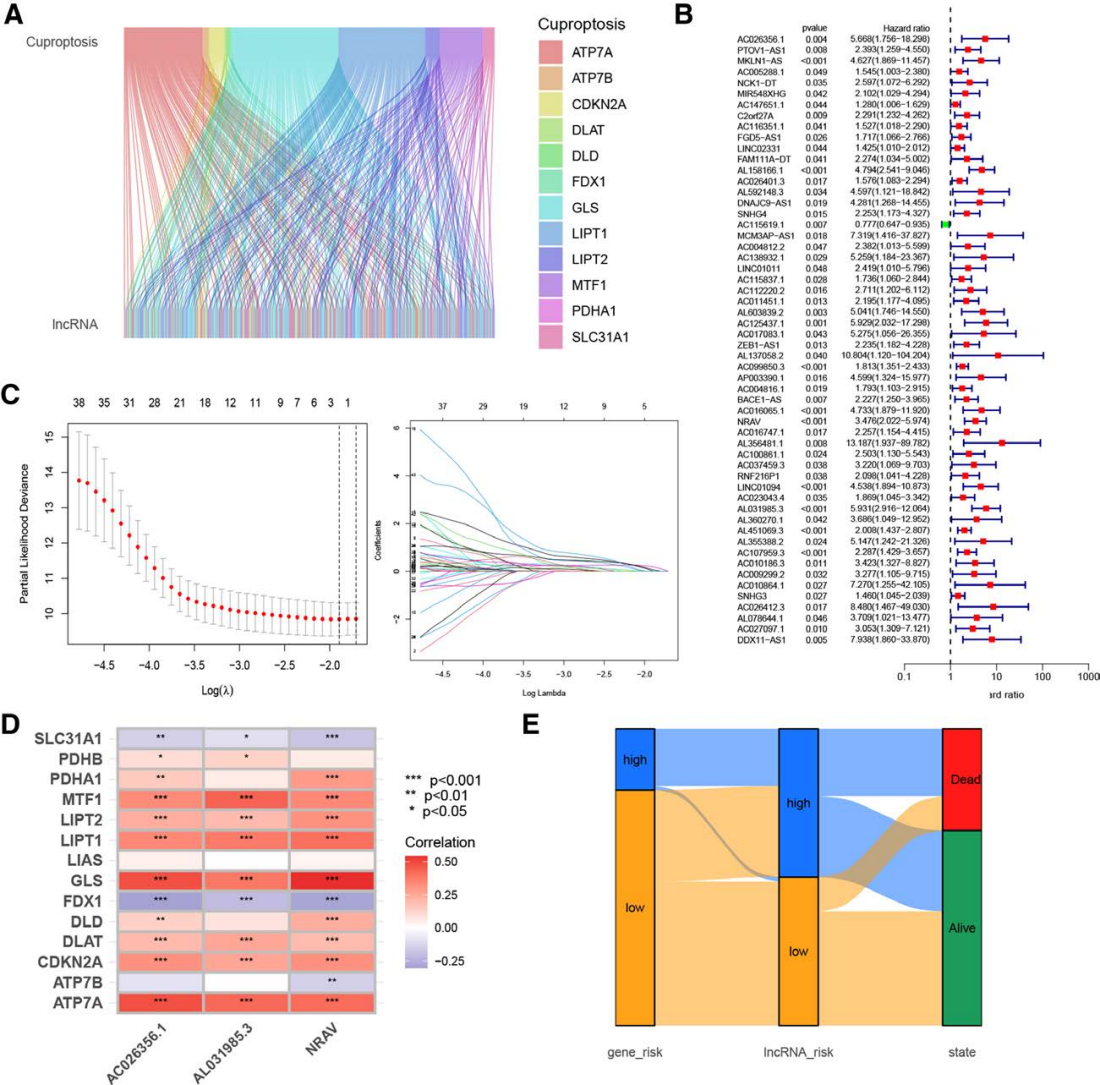
Cr-lncRNAs in HCC. (A) Univariate Cox regression analysis revealed the prognosis-related Cr-lncRNA. (B) Forest plot of univariate Cox regression analysis of 17 Cr-lncRNAs in the training set. (C) Optimal parameter (lambda) selection in the LASSO model. (D) The correlation between CRGs and risk Cr-lncRNAs. (E) The correlation between CRGs signature model and Cr-lncRNAs signature model. CRGs = cuproptosis-related genes, HCC = hepatocellular carcinoma.

Next, 291 CRGs-related lncRNAs were obtained in HCC from the TCGA dataset. The relationship between CRGs and Cr-lncRNAs was shown in Figure 5A. Among them, a univariate Cox regression analysis showed that 43 CRGs-related lncRNAs were correlated with survival in patients with HCC (Fig. 5B) and LASSO regression was used to construct a Cr-lncRNA prognosis model based on 3 key Cr-lncRNAs (AC026356.1, NRAV, AL031985.3) (Fig. 5C) in the training set. Figure 5D showed the correlation between CRGs and the risk of Cr-lncRNA. Figure 5E showed the correlation between the CRGs signature model, Cr-lncRNA signature model, and survival status.

Risk score = (0.599 * NRAV) + (0.848*AC026356.1) + (1.007* AL031985.3)

### 3.6. Evaluation and validation of Cr-lncRNAs signature in HCC

**Figure 6.**
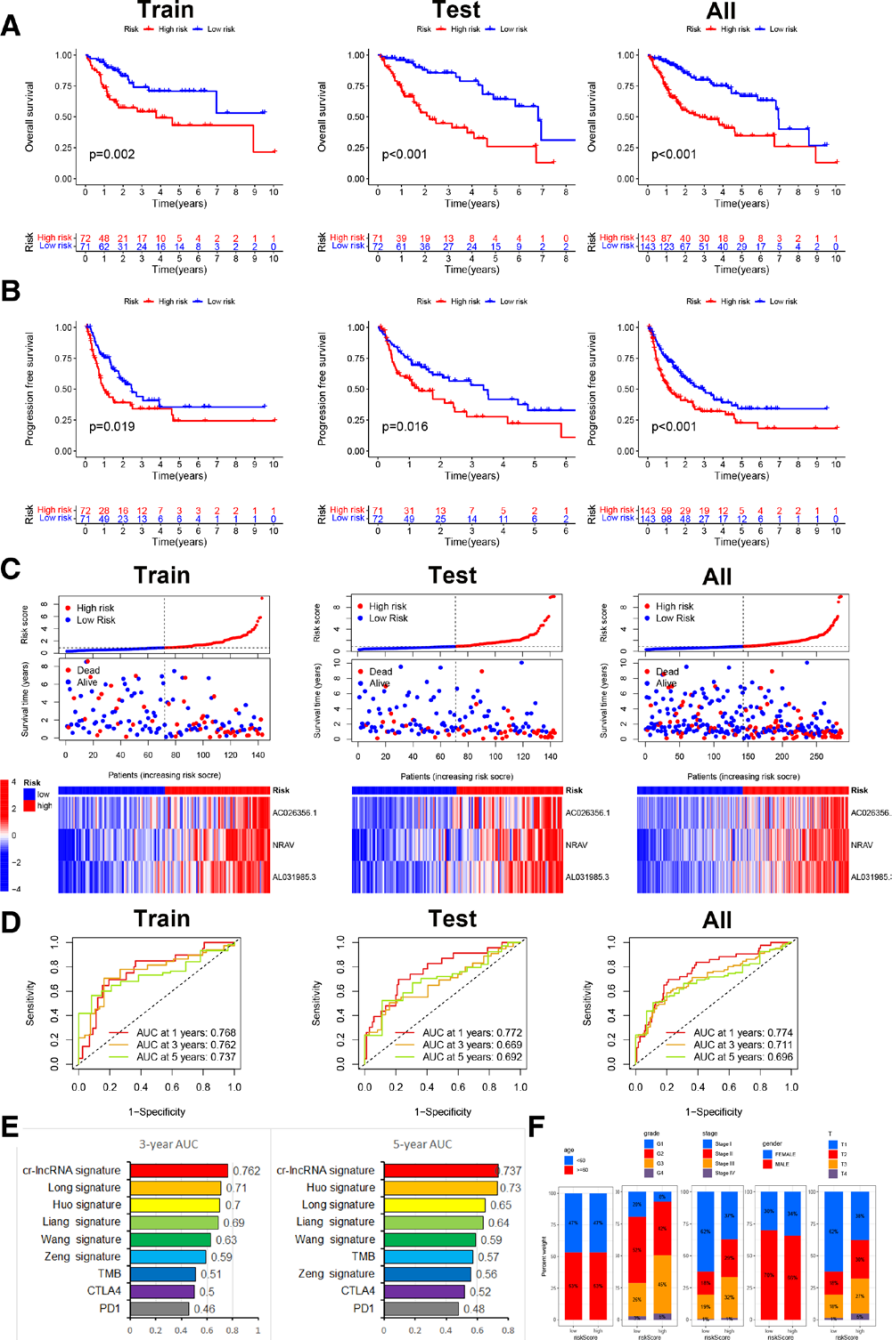
Construction, evaluation, and comparison of a risk signature. (A) Overall survival analysis of Cr-lncRNAs prognostic signature. (B) The progression free survival analysis of Cr-lncRNAs prognostic signature. (C) Risk score, survival status and heatmap of each HCC patient in the training, testing, and total sets. (D) ROC curve analysis of Cr-lncRNAs signature within 1, 3, and 5 yr. (E) ROC curve of Cr-lncRNA signature, several published signatures and biomarkers for 3- and 5-yr survival. (F) Correlation analysis between Cr-lncRNAs signature and clinical characteristic. Cr-lncRNAs = cuproptosis-related lncRNAs, HCC = hepatocellular carcinoma.

The predictive power of the prognostic model was assessed using multiple database analyses. Figure 6A exhibited that hepatoma carcinoma patients with high Cr-lncRNAs risk had poor OS in the training, testing, and total sets. Figure 6B showed that HCC patients with high Cr-lncRNAs risk had poor progression free survival (PFS) in the training, testing, and total sets. Scatter plots and risk curves showed the survival status and risk score of each HCC patient in the training, testing, and total sets (Fig. 6C). The predictive value of the CRGs signature in the training was analyzed by ROC curve (1-year AUC = 0.768, 3-year AUC = 0.762, 5-year AUC = 0.737), testing (1-year AUC = 0.772, 3-year AUC = 0.669, 5-year AUC = 0.692), and total sets (1-year AUC = 0.774, 3-year AUC = 0.711, 5-year AUC = 0.696) (Fig. 6D). As shown in Figure 6E, Cr-lncRNAs had the highest AUC for either 3-year (AUC = 0.762) or 5-year survival (AUC = 0.737), compared to several signatures (Long signature, Huo signature, Liang signature, Wang signature, and Zeng signature) and biomarkers (TMB, CTLA4, and PD1). Correlation analysis between the Cr-lncRNAs signature and clinical characteristics revealed that the risk score was markedly associated with HCC grade and stag (Fig. 6F). The above results fully illustrated that the Cr-lncRNAs signature was dependable signature.

PCA analysis was utilized to differentiate between high-risk and low-risk groups based on IncRNA, cuproptosis-related IncRNAs, and all genes (see Fig. S6A, http://links.lww.com/MD/J510, Supplemental Content, which illustrates the PCA analysis of genes in Cr-lncRNAs high/low risk group), and the results revealed that the distributions of the high-risk and low-risk groups were well distinguished. Moreover, the waterfall plot showed that high-risk patients had more P53 mutations (see Fig. S6B, http://links.lww.com/MD/J510, Supplemental Content, which illustrates the waterfall plot of mutation analysis). Tumor mutation burden analysis indicated that the H-TMB + high risk group had a poor survival probability (see Fig. S6C, http://links.lww.com/MD/J510, Supplemental Content, which illustrates the tumor mutation burden of HCC patients in Cr-lncRNAs high-/low- risk group).

### 3.7. Prognosis of HCC is independent of Cr-lncRNAs signature

**Figure 7.**
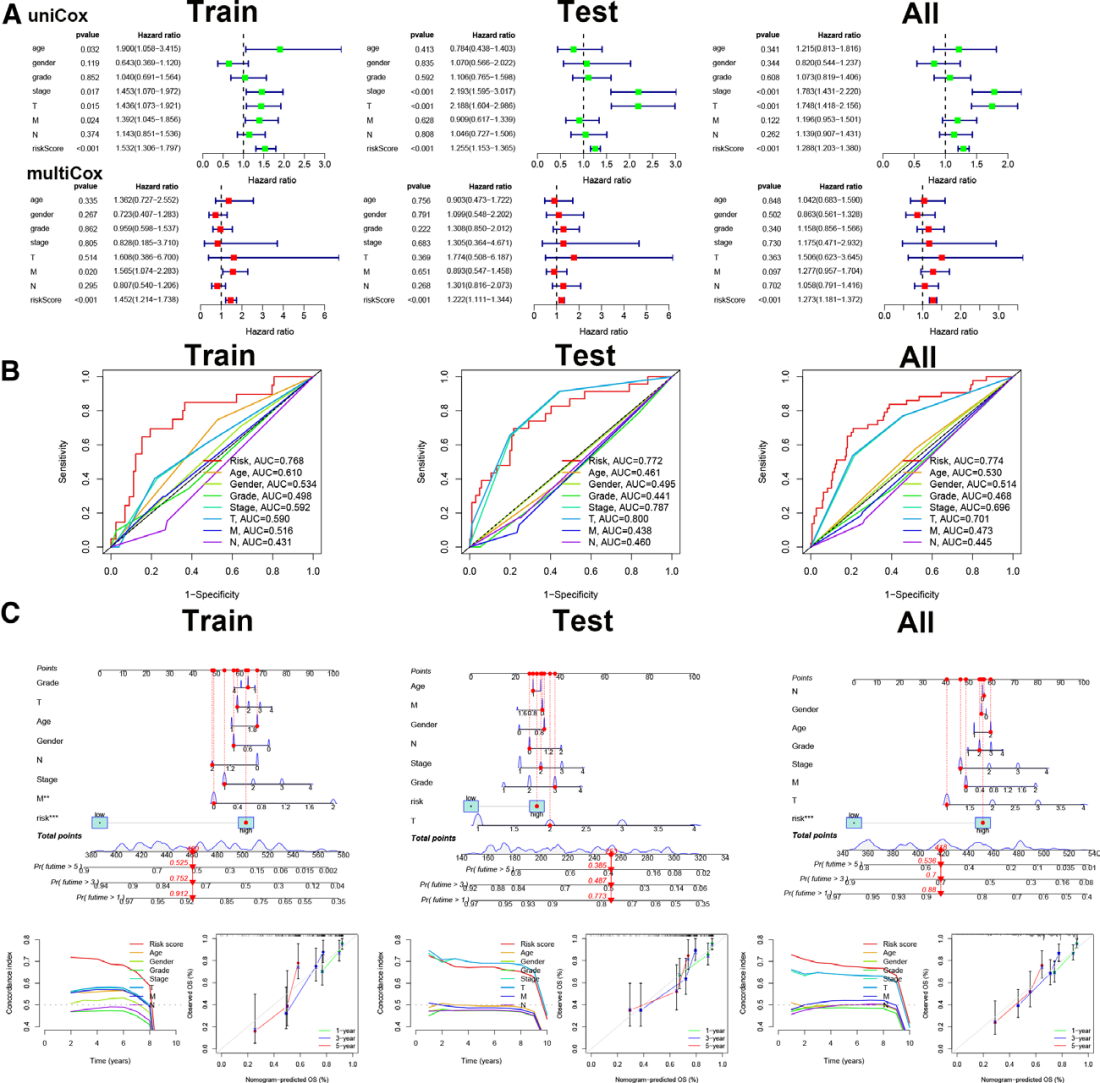
Cr-lncRNAs signature as an independent prognostic factor for HCC. (A) The univariate and multivariate analysis for the training, testing, and total sets. (B) Cr-lncRNAs prognostic signature compared with other clinical indicators by multivariate ROC curve analysis in the train, test, and total sets. (C) The nomogram model and calibration plots for predicted probability in the train, test, and total sets. Cr-lncRNAs = cuproptosis-related lncRNAs, HCC = hepatocellular carcinoma.

Next, we conducted univariate and multivariate analyses to evaluate whether Cr-lncRNAs could be independent prognostic factors for patients with HCC. Figure 7A showed that the HR of Cr-lncRNAs risk score was 1.532 (95%CI: 1.306–1.797) and 1.452 (95%CI: 1.214–1.738) in the univariate and multivariate analyze, respectively, indicating that it was an independent prognostic factor in HCC using training, testing, and total sets. Figure 7B showed that the Riskscore (AUC = 0.768, AUC = 0.772, and AUC = 0.774) was assessed considerably well compared with an ideal model utilizing training, testing, and total sets, respectively. The risk score and clinicopathologic features were then collected to build a predictive nomogram, suggesting that the risk score in the 3 datasets could better predict the prognosis of HCC (Fig. 7C).

### 3.8. Cr-lncRNAs signature is associated with immune microenvironment in HCC

**Figure 8.**
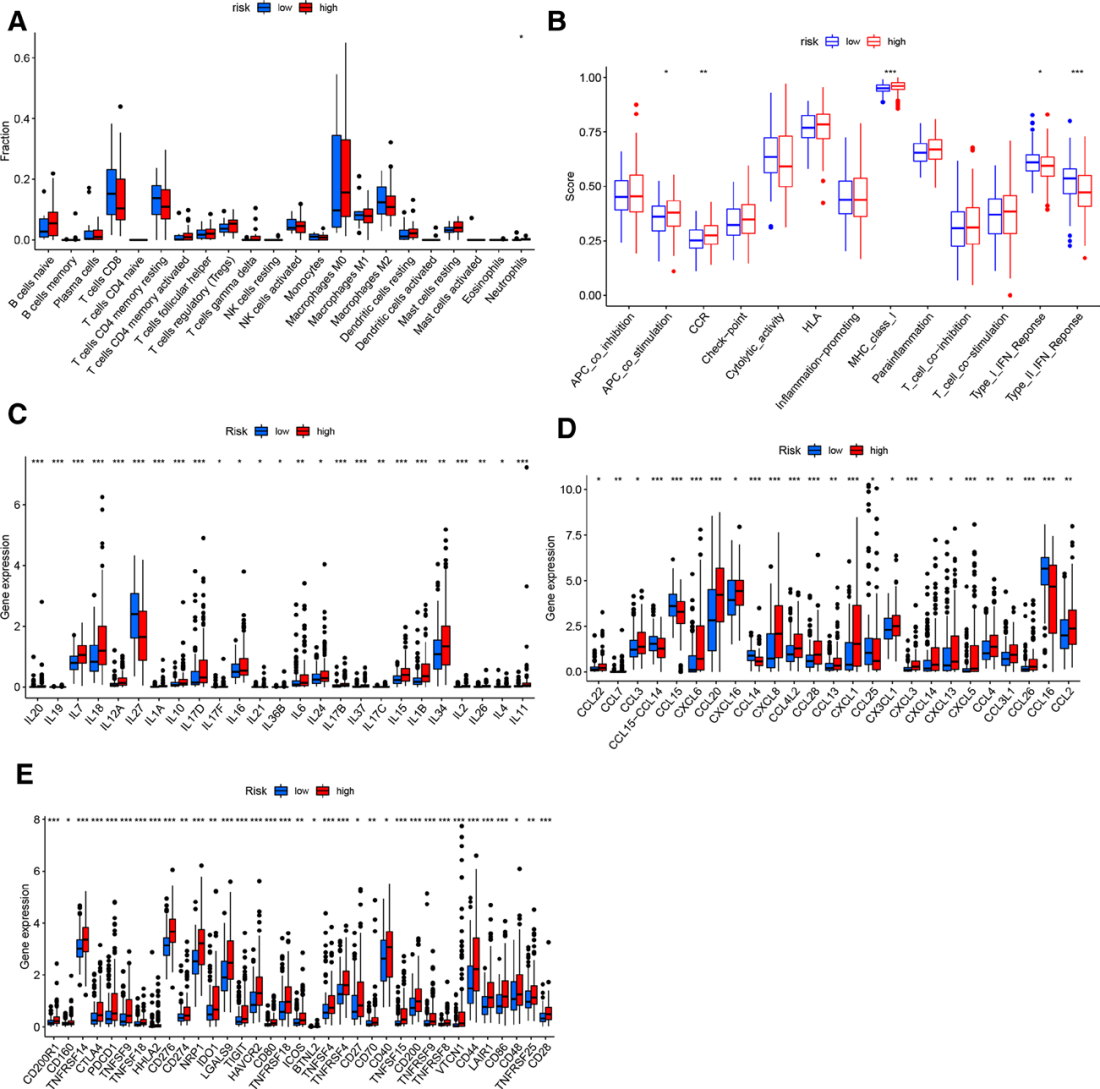
The correlation between Cr-lncRNA signature and immune microenvironment in HCC. The comparable score of immune cells (A) and functions (B) based on the prognostic risk model. Cytokines (C) and chemokines (D), checkpoint (E) expression in high/low risk group. Cr-lncRNAs = cuproptosis-related lncRNAs, HCC = hepatocellular carcinoma.

Based on the ssGSEAScore in TCGA data, we analyzed the tumor immune microenvironment between HCC patients and high-/low- risk patients. Mast cells resting and T cells regulatory, etc were activated in high-risk patients (Fig. 8A). Immune functions, including APC_co_stimulation, CCR, and MHC_class_I, were over-expressed in high-risk patients. The type_ II_IFN_Response and type_I_IFN_Response were higher in low-risk patients (Fig. 8B). Next, we analyzed cytokines and chemokines expression in the high- and low-risk groups. As shown in Figure 8C, the expression levels of IL7, IL18, IL12A, IL10, IL17D, IL16, IL6, IL24, IL15, IL1B, and IL34 were higher in the high-risk group. The expression of CCL22, CCL3, CXCL6, CCL20, CXCL8, CCL4L2, CCL13, CXCL1, CX3CL1, CXCL3, CXCL14, CXCL13, CXCL5, CCL4, CCL3L1, CCL26, and CCL2 increased in the high-risk group (Fig. 8D). We then analyzed checkpoint expression in HCC with high/low Cr-lncRNA risk scores. As shown in Figure 8E, the expression of the 35 checkpoints was higher in Cr-lncRNAs high-risk patients than in Cr-lncRNAs low-risk patients.

### 3.9. Drug sensitive of CRGs signature and Cr-lncRNAs signature in HCC

**Figure 9.**
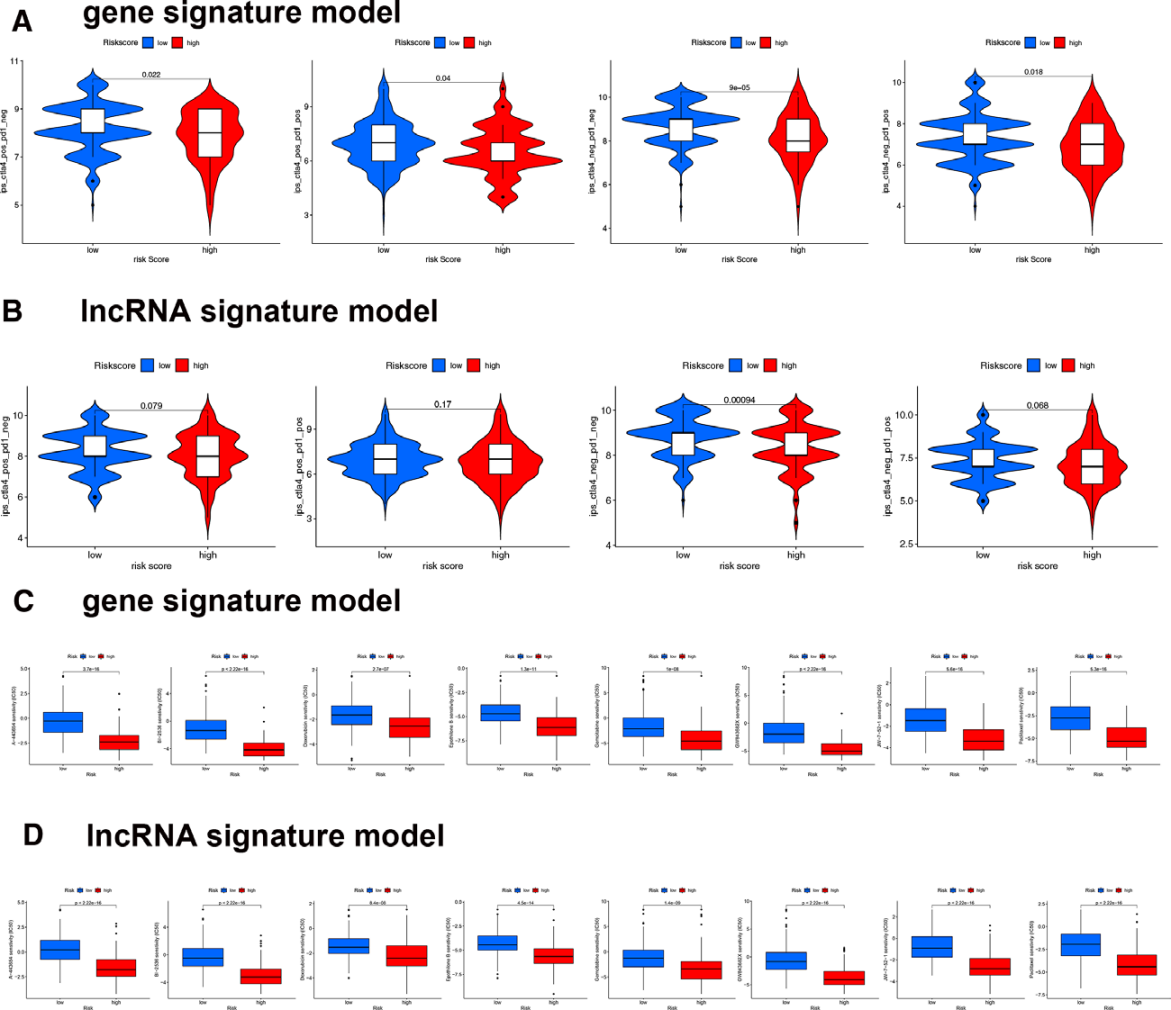
Drug-sensitivity analysis of CRGs and Cr-lncRNA in HCC. IPS in different risk score groups in gene risk model (A) and lncRNA signature model (B). Differential chemotherapeutic responses in high- and low-risk patients in gene signature model (C) and lncRNA signature model (D). CRGs = cuproptosis-related genes, Cr-lncRNAs = cuproptosis-related lncRNAs, HCC = hepatocellular carcinoma, IPS = immunophenoscore.

To further study drug responses in patients, we evaluated the immunophenoscore scores to evaluate the immune response of HCC patients using gene and lncRNA signature models. Figure 9A showed that high-risk patients had a higher immunophenoscore score, implying that patients in the low-risk group may have greater sensitivity to immunotherapy in the gene risk model. However, there was no significant sensitivity to immunotherapy in the lncRNAs signature model between low- and high-risk patients (Fig. 9B). Moreover, 8 drugs (A-443654, BI-2536, doxorubicin, epothilone B, gemcitabine, GW843682X, JW-7-52-1, and Paclitaxel) showed distinct estimated IC_50_ values between high- and low-risk HCC patients in the gene risk model (Fig. 9C) and lncRNA signature mode (Fig. 9D), and the results indicated that patients with high risk may respond better to these drugs (see Fig. S7A and 7B, http://links.lww.com/MD/J511, Supplemental Content, which illustrates the correlation with risk score and chemotherapeutic sensitivity in HCC).

## 4. Discussion

HCC is the most common malignant liver cancer, with a strong ability to invade and metastasize. In recent years, an increasing number of studies have shown that genes and lncRNAs expression may be related to tumorigenesis and the progression of tumors, suggesting that genes and lncRNAs are expected to be novel biomarkers and therapeutic targets.^[11–15]^ Cuproptosis is a unique proteotoxic cell death pathway in which copper binds directly to the lipoylated proteins of the tricarboxylic acid cycle cycle.^[5]^ However, the expression and roles of CRGs and lncRNAs in HCC remain unclear. In this study, CRGs and Cr-lncRNAs signatures were constructed and verified based on 4 CRGs and 3 Cr-lncRNAs, respectively.

At the beginning of the study, we screened out 14 the expression and variation of genes related to cuproptosis in HCC, and the expression of 13 CRGs was found to be differentially expressed in HCC tissues compared with normal tissues. In HCC patients, 11 were related to OS in both the TCGA and ICGC training sets. The CRGs model was constructed based on 4 CRGs (ATP7A, MTF1, GLS, and CDKN2A) in patients with HCC. ATP7A, a copper efflux transporter, is critical for the delivery of copper to physiological processes, whereas Menkes and Wilson diseases are accompanied by dysregulation of ATP7A function.^[16]^ In our study, we found that ATP7A expression was higher in HCC tissues than in adjacent normal tissues, and it was a risk factor for HCC prognosis. Based on the present results, previous studies have consistently demonstrated that low ATP7A expression could result in better survival, as well as enhanced chemotherapeutic response in patients with cancer, including platinum drug resistance.^[17,18]^ Elesclomol-induced copper chelation inhibited colorectal cancer by targeting ATP7A,^[19]^ suggesting that ATP7A may be a potential drug treatment target. MTF1, a transcription factor, maintains copper homeostasis and protects cells against injury caused by excess copper.^[20]^ GLS plays a crucial role in the regulation of glutamine metabolism, and has been recognized as a novel biomarker and therapeutic target in cancers, such as breast cancer^[21]^ and prostate cancer.^[22]^ Furthermore, GLS is a CRG in renal cell carcinoma.^[10]^ In our study, CDKN2A possessed the most frequent mutations. Consist with our studies, multiple studies have revealed that genomic alterations in CDKN2A was the most commonly phenomenon in cancers,^[23,24]^ including neck cancers (50%), pancreatic and esophageal cancers (47%), gliomas (35%), implying the correlation between CDKN2A alterations and tumors. Moreover, CDKN2A, a CRG,^[10]^ participates in various cellular pathways, including inhibiting tumor cell apoptosis and enhancing tumor cell proliferation.^[25]^ In addition, high expression of CDKN2A can affect the prognosis of patients by regulating tumor-associated macrophages.^[26]^ In this study, the CRGs model was an independent prognostic factor for HCC with high prognostic accuracy.

lncRNAs participated in the occurrence, progression and prognosis of tumors by regulating gene expression.^[27–29]^ In this study, 43 Cr-lncRNAs were obtained and associated with the survival of HCC using Cox proportional hazards analysis, 3 key Cr-lncRNAs, including AC026356.1, NRAV, and AL031985.3, were obtained by Lasso regression, and a prognostic model was established. The role of AC026356.1 in cancer has not yet been validated by other studies, suggesting AC026356.1 may be involved in copoptosis and a novel prognostic biomarker in HCC. The function of NRAV is related to immune checkpoints,^[30]^ ferroptosis,^[31]^ and activation of Wnt/β-catenin signaling through regulation of the miR-199a-3p/CISD2 axis,^[32]^ implying that NRAV may participate not only in copoptosis but also in activating other signaling pathways to perform its function in the tumorigenesis of HCC. Some studies have reported that lncRNA AL031985.3 has prognostic value in HCC,^[33–35]^ which is consistent with our study. In this study, we identified 3 key Cr-lncRNAs and constructed risk and prognostic models. Moreover, high-risk patients had poorer OS and poor progression free survival than low-risk patients, and the risk score was significantly associated with Cr-lncRNAs and could be an independent Cr-lncRNAs prognostic factor for HCC.

While immune microenvironment consists of immune cells, cytokines, and chemokines, which play vital roles in cancer progression.^[36]^ A growing number of studies have demonstrated that tumor-infiltrating lymphocytes drive immune evasion during HCC progression, including regulatory T cells, tumor-associated macrophages, etc.^[37]^ Previous study found that Macrophages M0 and T cells regulatory played a tumor-promoting role in Papillary Thyroid Carcinoma.^[38]^ In our study, increased immune cells infiltration, including Macrophages M0 and Mast cells resting, were found in the high- risk group of the CRGs model. T cells regulatory, etc, evidently increased in the high-risk patients of the Cr-lncRNAs model. High-risk HCC patients had high levels of M0 macrophages, and regulatory T cells, which indicated immune tolerance. Although a previous study reported that copper plays an important role in the maintenance of immunocompetence,^[39]^ the immune molecular regulation of the tumor microenvironment remains unclear. Immune function indicated that the Type II IFN response was meaningfully lower in high-risk patients than in low-risk patients. In addition, immune infiltration analysis demonstrated the differential expression of cytokines and chemokines associated with the CRGs and Cr-lncRNAs models. These results implied that the microenvironments, respectively antitumor immune response involved in CRGs and Cr-lncRNAs-mediated HCC prognosis.

Anticancer immunotherapies involving immune-checkpoint inhibitors have become a novel and effective therapeutic strategy. Immune-checkpoint inhibitors, such as anti-PD1/PD-L1 and anti-CTLA4, have been shown to activate the host immune response and have been approved for the treatment of multiple types of cancer.^[40–43]^ The expression of immune checkpoints increased in the high-risk group of both the CRGs and Cr-lncRNAs models. In our study, drug sensitivity indicated that patients with low-risk of both CRG signature and Cr-lncRNA signature showed positive responsiveness for anti-PD1 and anti-CTLA-4 therapy in the gene signature model, and 8 drugs represented more sensitivity for patients at high-risk in the gene signature model and lncRNA signature model. These results offered the foothold for probing novel immunotherapy strategies between low-risk and high-risk groups in HCC patients. However, drug mechanisms and their impact on HCC progression should be the focus of further studies.

## 5. Conclusions

Overall, this is the first report to screen the CRGs and Cr-lncRNAs in HCC, construct a CRGs and Cr-lncRNAs signature model for prognostic biomarkers, and explore the immune microenvironment and therapeutic response of HCC patients. This work provides an innovative tool for diagnosis and a new basis for immunotherapy of HCC and has the potential to guide individualized healthcare decisions in the future.

## Author contributions

**Conceptualization:** Hong Peng, Yiya Zhang, Xiaozhen Peng.

**Data curation:** Zhaoxia Zou, Xingjun Lu.

**Formal analysis:** Hong Peng, Zhaoxia Zou, Ziye Xiang, Yiya Zhang.

**Methodology:** Zhaoxia Zou, Yiya Zhang, Xiaozhen Peng.

**Software:** Yiya Zhang, Xiaozhen Peng.

**Visualization:** Hong Peng, Yiya Zhang, Xiaozhen Peng.

**Writing – original draft:** Hong Peng, Yiya Zhang, Xiaozhen Peng.

**Writing – review & editing:** Xiaozhen Peng.

## Supplementary Material














