# Supplementary material for: Cuproptosis-related prognostic signatures predict the prognosis and immunotherapy in HCC patients
Source: Medicine (Baltimore). 2023 Aug 25;102(34):e34741. doi: 10.1097/MD.0000000000034741 (PMC10470811; doi:10.1097/MD.0000000000034741)

Supplemental Digital Content. Figure S1. Selection of CRGs in HCC. (A) The heatmap of CRGs expression in HCC. (B) The variation of CRGs in HCC from TCGA. (C) The CNV values of CRGs in HCC from TCGA. (D) The location of CRGs.

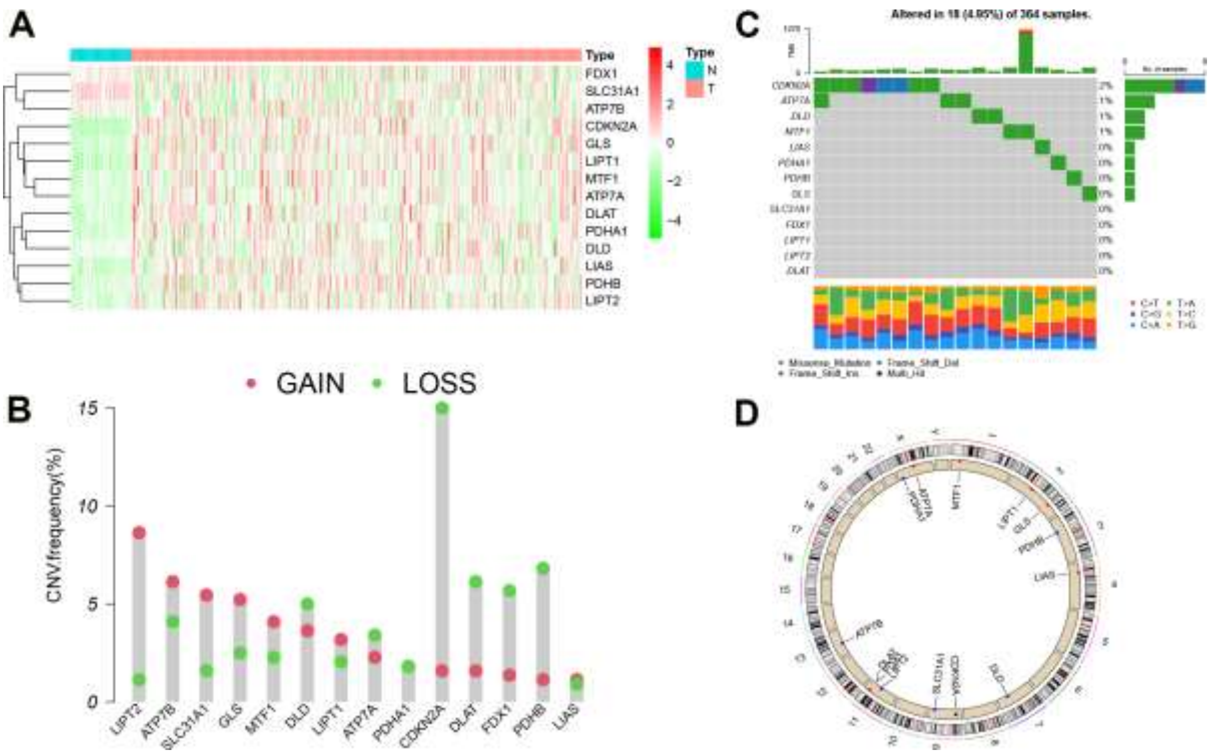

Supplement: Supplementary file 1 [file medi-102-e34741-s001.pdf]
