# Supplementary figures and images for: Cuproptosis-related prognostic signatures predict the prognosis and immunotherapy in HCC patients
Source: Medicine (Baltimore). 2023 Aug 25;102(34):e34741. doi: 10.1097/MD.0000000000034741 (PMC10470811; doi:10.1097/MD.0000000000034741)

Supplemental Digital Content. Figure S2. GSVA analysis.

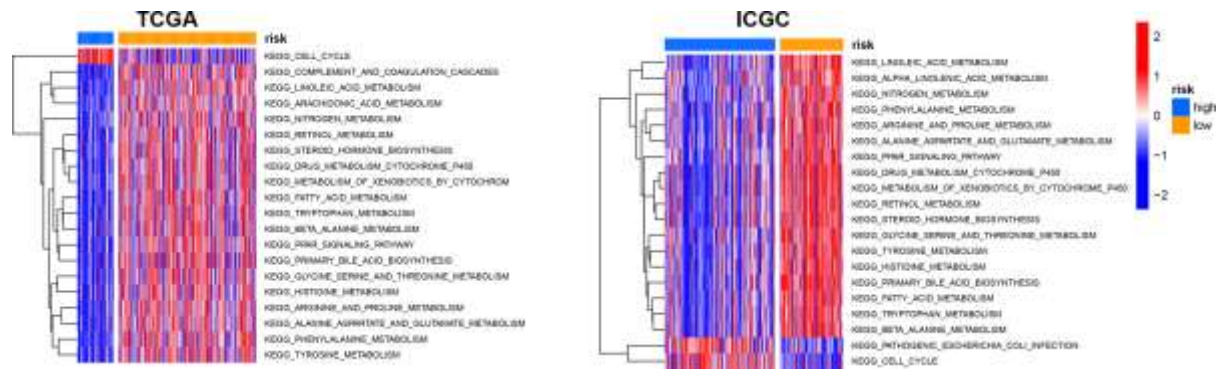

Supplement: Supplementary file 2 [file medi-102-e34741-s002.pdf]

Supplemental Digital Content. Figure S5. The different immune infiltration in high/low group.

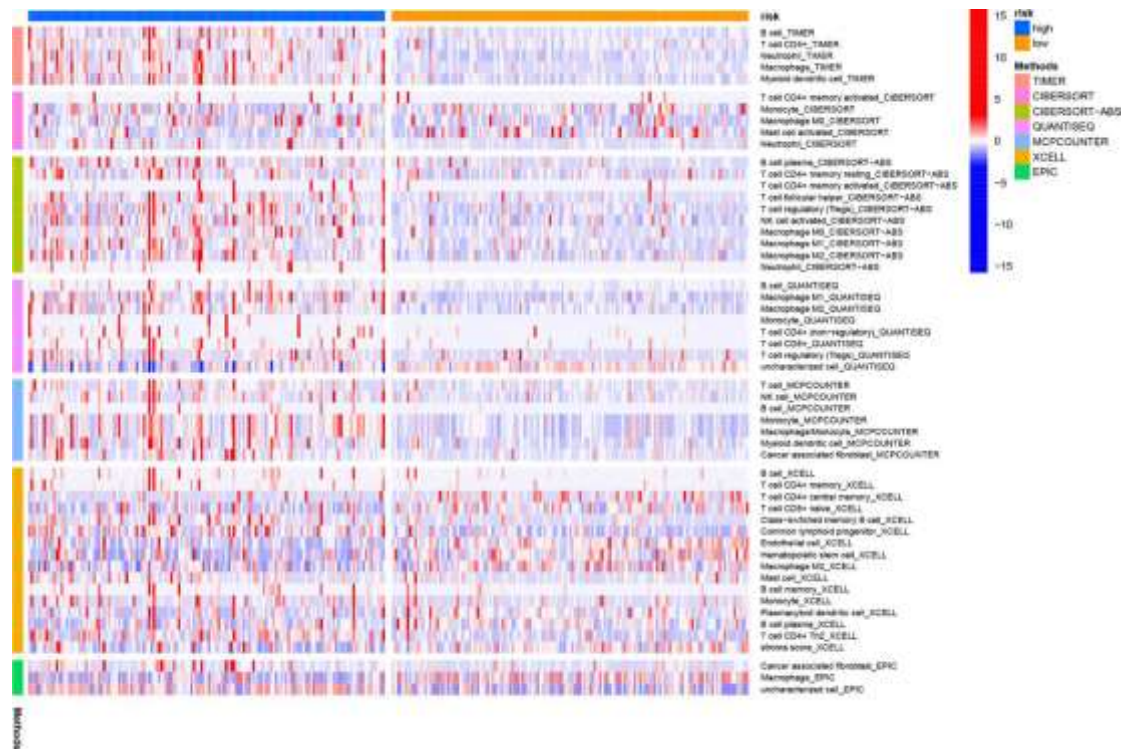

Supplement: Supplementary file 5 [file medi-102-e34741-s005.pdf]
