# Supplementary material for: Cuproptosis-related prognostic signatures predict the prognosis and immunotherapy in HCC patients
Source: Medicine (Baltimore). 2023 Aug 25;102(34):e34741. doi: 10.1097/MD.0000000000034741 (PMC10470811; doi:10.1097/MD.0000000000034741)

Supplemental Digital Content. Figure S3. The survival analysis of HCC patients with high/low risk.

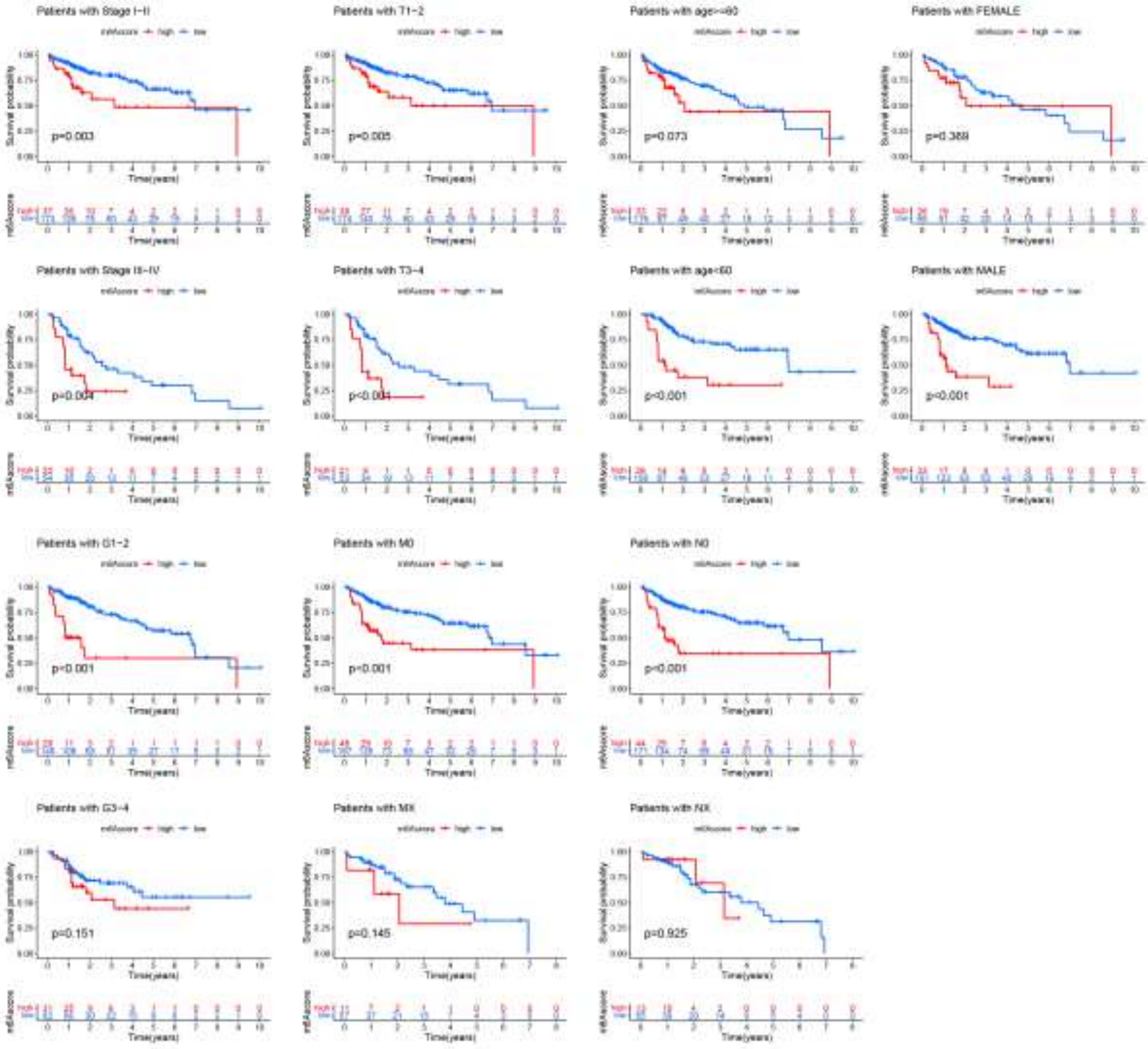

Supplement: Supplementary file 3 [file medi-102-e34741-s003.pdf]
