# Supplementary material for: Cuproptosis-related prognostic signatures predict the prognosis and immunotherapy in HCC patients
Source: Medicine (Baltimore). 2023 Aug 25;102(34):e34741. doi: 10.1097/MD.0000000000034741 (PMC10470811; doi:10.1097/MD.0000000000034741)

Supplemental Digital Content. Figure S4. The gene variation of HCC patients in high/low risk. The tumor mutation burden of patient of HCC in high/low risk group (A) and (B).

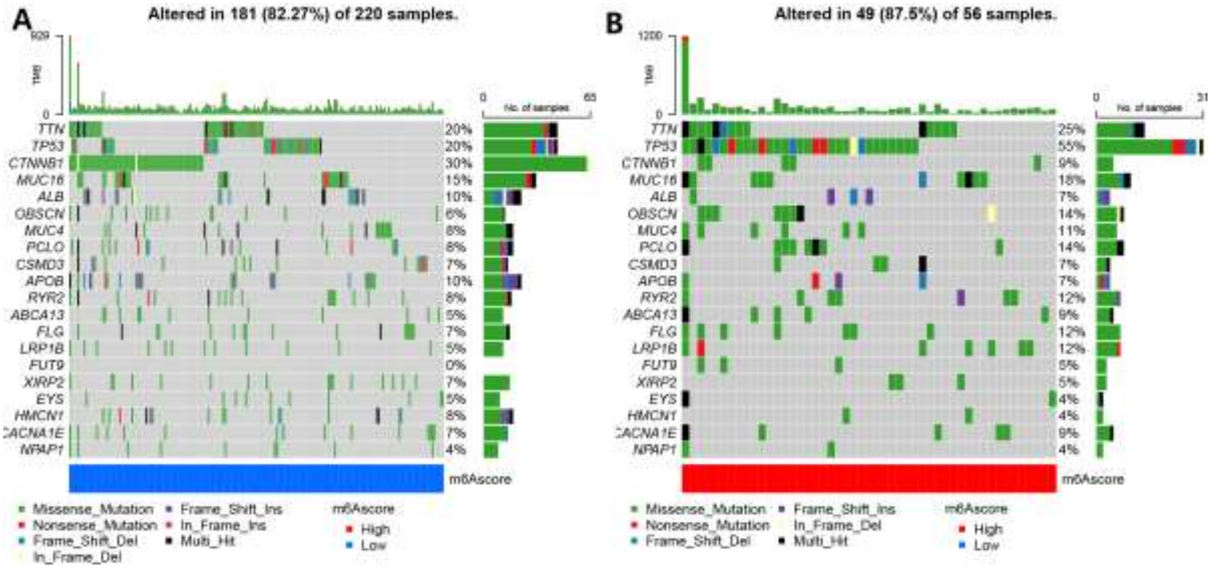

Supplement: Supplementary file 4 [file medi-102-e34741-s004.pdf]
