# Supplementary material for: Cuproptosis-related prognostic signatures predict the prognosis and immunotherapy in HCC patients
Source: Medicine (Baltimore). 2023 Aug 25;102(34):e34741. doi: 10.1097/MD.0000000000034741 (PMC10470811; doi:10.1097/MD.0000000000034741)

Supplemental Digital Content. Figure S6. The gene variation of HCC patients in high-/low-risk. (A) The PCA analysis of genes in Cr-lncRNAs high/low risk group. (B) The waterfall plot of mutation analysis. (C) The tumor mutation burden of HCC patients in Cr-lncRNAs high-/low- risk group.

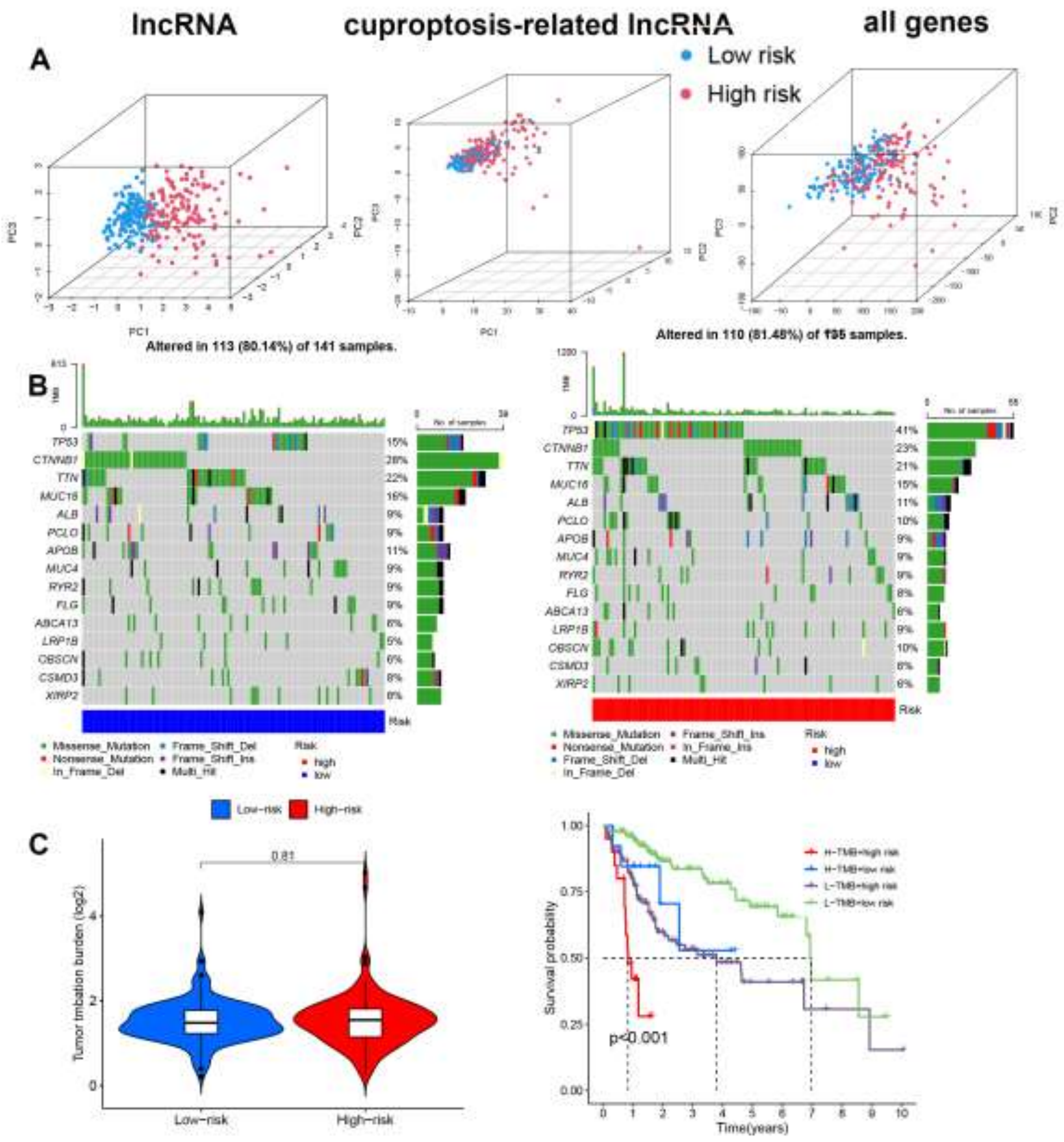

Supplement: Supplementary file 6 [file medi-102-e34741-s006.pdf]
