# Supplementary material for: Cuproptosis-related prognostic signatures predict the prognosis and immunotherapy in HCC patients
Source: Medicine (Baltimore). 2023 Aug 25;102(34):e34741. doi: 10.1097/MD.0000000000034741 (PMC10470811; doi:10.1097/MD.0000000000034741)

Supplemental Digital Content. Figure S7. Risk score was associated with chemotherapeutic sensitivity in HCC. Relationships between drugs sensitivity and risk score in gene signature model (A) and lncRNA signature model (B).

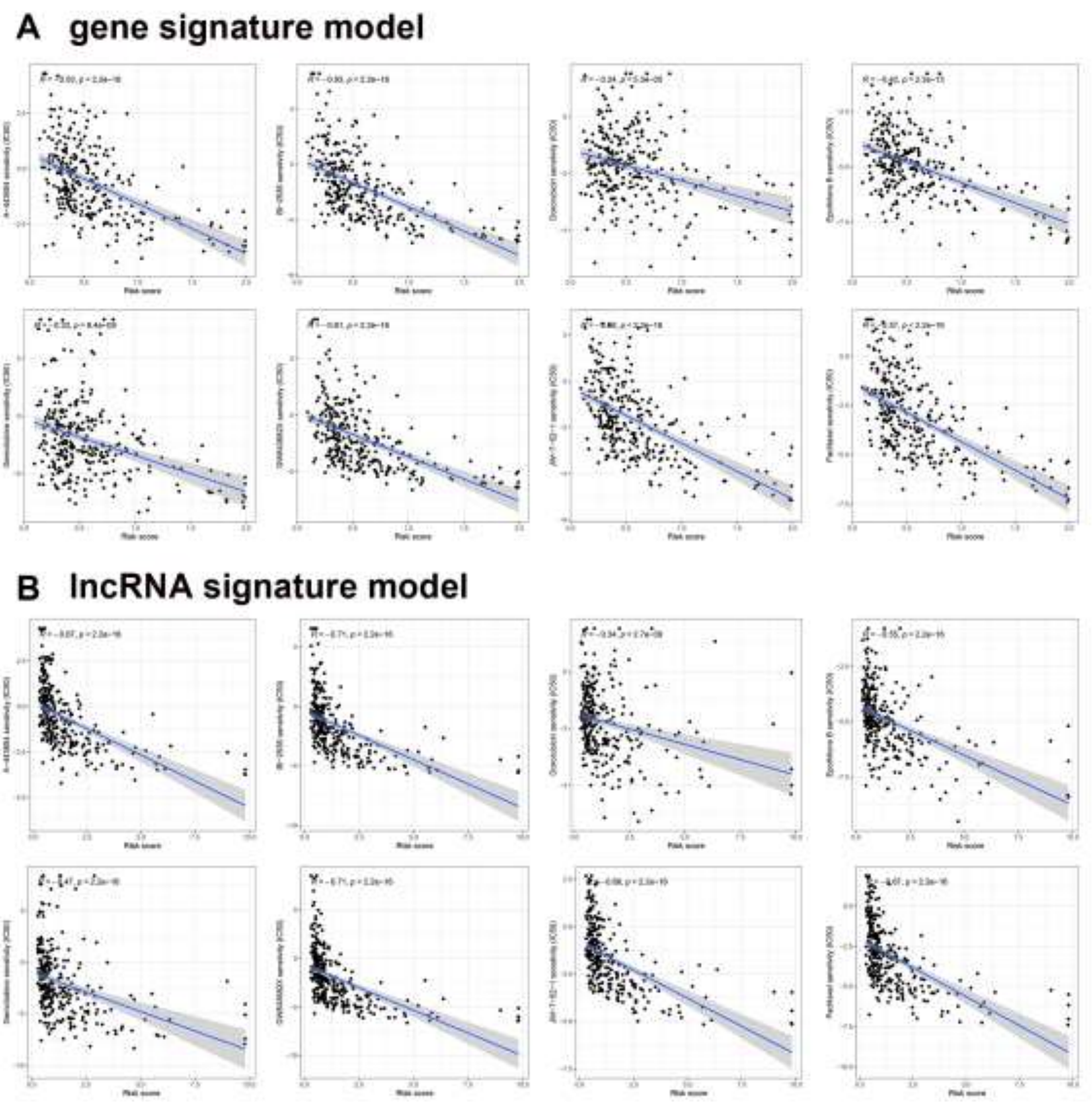

Supplement: Supplementary file 7 [file medi-102-e34741-s007.pdf]
